# Modulation of stretch activation influences the stretch‐shortening cycle effect in in vivo human knee extensors

**DOI:** 10.14814/phy2.70377

**Published:** 2025-05-15

**Authors:** Iseul Jo, Wolfgang Seiberl, Hae‐Dong Lee

**Affiliations:** ^1^ Department of Physical Education, Graduate School Yonsei University Seoul Korea; ^2^ Frontier Research Institute of Convergence Sports Science, College of Educational Sciences Yonsei University Seoul Korea; ^3^ Institute of Sports Science University of the Bundeswehr Munich Neubiberg Germany; ^4^ Department of Physical Education, College of Educational Sciences Yonsei University Seoul Korea

**Keywords:** fascicle, force depression, muscle‐tendon dynamics, stretch‐shortening cycle, ultrasonography

## Abstract

This study investigated the effects of progressively increasing voluntary activation during the stretch phase on force and work production in the stretch‐shortening cycle (SSC) of human knee extensors. Fifteen young adults performed SSCs under four stretch activation conditions: passive stretch (ST_0%_‐SC), feedback‐guided active stretch (ST_40%_‐SC and ST_80%_‐SC), and maximal effort stretch (ST_100%_‐SC). All conditions involved maximal voluntary activation during shortening, followed by a fixed‐end contraction at 20°. Outcome measures included joint torque and work, estimated fascicle force and work, vastus lateralis fascicle length and velocity, and quadriceps activation. Compared to passive stretch, active stretch conditions produced greater SSC effects, with no significant differences between ST_80%_‐SC and ST_100%_‐SC. Fascicle work did not differ significantly across conditions, suggesting a decoupling between joint‐level output and fascicle‐level contribution. Active stretch primarily enhanced force production during early shortening; however, the SSC effect persisted until mid‐to‐late shortening (80° to 38°) in ST_80%_‐SC. ST_0%_‐SC also showed nearly twice the fascicle shortening velocity of other conditions. Following shortening, ST_100%_‐SC exhibited greater residual force depression during the isometric phase, despite similar activation. These findings demonstrate that voluntary activation during stretch modulates SSC effect through a complex interplay involving muscle‐tendon unit decoupling and history‐dependent effects, fascicle dynamics, and tendon compliance.

## 1 INTRODUCTION

The stretch‐shortening cycle (SSC) represents a fundamental muscle‐tendon unit (MTU) behavior observed in various human movements (Komi, 1984, 2000). It involves an eccentric action (MTU lengthening) immediately followed by a concentric action (MTU shortening). This behavior has drawn the attention of scientists because of the enhanced mechanical force and work output during the shortening phase of SSC compared to shortening without prior active lengthening. This phenomenon, referred to as the SSC effect, has been consistently observed in studies with various experimental preparations (Cavagna et al., 1968; Joumaa et al., 2008; Leonard et al., 2010; Nikolaidou et al., 2017).

With the wealth of information on the SSC effect, its underlying mechanisms have been predominantly attributed to neuromuscular dynamics, such as pre‐activation (Bobbert & Casius, 2005; Svantesson et al., 1994) and stretch reflex responses (Dietz et al., 1979; van Ingen Schenau et al., 1997a, 1997b), which facilitate immediate force production during the early shortening phase, and (Komi, 2000) muscle‐tendon unit (MTU) dynamics, including the utilization of stored elastic energy (Alexander & Bennet‐Clark, 1977; Farris et al., 2016; Finni et al., 2001; Kawakami et al., 2002). However, experimental and simulation studies (Ettema et al., 1992; Walshe et al., 1998) have suggested that neuromuscular and elastic mechanisms alone may be insufficient to fully explain the SSC effect, and that potentiation within the contractile element may also contribute to enhanced force production.

Recent advancements over the past decade have identified two significant contributors: the residual force enhancement (rFE) effect mediated by titin, an intrasarcomeric molecular spring (Hahn & Riedel, 2018; Seiberl et al., 2015; Tomalka et al., 2020), and MTU decoupling, which increases force capacity during shortening by reducing fascicle shortening velocity following stretch (Holzer et al., 2024). For example, Seiberl et al. (2015) demonstrated that the rFE persists during SSC in human adductor pollicis using electrically evoked contractions. Groeber et al. (2020) investigated the quadriceps femoris and showed that the type of activation, whether maximal voluntary or submaximal electrical, modulates the magnitude of the SSC effect. Groeber et al. (2021) demonstrated that rFE effects are independent of stretch magnitude but influenced by where on the torque‐angle relationship the stretch was applied. Furthermore, Holzer et al. (2024) explored fascicle behavior during SSC in the triceps surae using electrical stimulation, underscoring the importance of MTU decoupling in SSC effects.

Significantly, rFE interacts with residual force depression (rFD), a reduction in steady‐state isometric force following muscle shortening (Maréchal & Plaghki, 1979). In situ studies have reported that rFE, generated during the stretching contraction, is negated during the subsequent shortening phase (Herzog & Leonard, 2000; Lee & Herzog, 2003). However, in vivo studies have reported conflicting results regarding the persistence of rFE. Some studies have observed sustained rFE in steady‐state isometric contractions (Fortuna et al., 2018; Hahn & Riedel, 2018; Seiberl et al., 2015), while others suggest that rFE is mitigated similarly to in situ findings (Fukutani et al., 2017; Groeber et al., 2021). These inconsistencies indicate no clear consensus on rFE behavior in vivo, unlike in in situ conditions.

Despite these advances, the applicability of these mechanisms to natural human movement remains unclear. Unlike controlled activation in experimental setups, voluntary movement involves gradual increases in muscle activation (Bohm et al., 2018; Nikolaidou et al., 2017). Additionally, many studies employed steady‐state isometric pre‐activation protocols (Fortuna et al., 2018; Groeber et al., 2021; Seiberl et al., 2015), which can alter fascicle length behavior during SSC phases (Fukutani et al., 2016a, 2016b). These methodological differences may lead to over‐ or underestimation of rFE effects compared to voluntary contractions observed in natural movements (Groeber et al., 2020; Jacob et al., 2022; Paternoster et al., 2021). Therefore, this study investigates the SSC effect in in vivo human knee extensors under voluntary activation patterns similar to those that occur during natural human movements. Specifically, we examine how a gradual increase in activation during the stretch phase influences torque, work, and fascicle behavior during the subsequent shortening phase.

## 2 MATERIALS AND METHODS

### 2.1 Participants

In this study, ten young adult males (age: 25.5 ± 2.7 years, height: 175.0 ± 3.2 cm, weight: 80.9 ± 8.4 kg) and five females (age: 26.0 ± 2.5 years, height: 162.3 ± 4.6 cm, weight: 59.2 ± 13.3 kg) were included. All participants were healthy individuals without a history of lower limb surgery in the past year. Before participation, they received detailed information about the study and provided written informed consent. The Yonsei University Institutional Review Board approved the study protocol (7001988‐202405‐HR‐2275‐02).

A priori power analysis was conducted using G*Power based on partial eta‐squared values reported in Groeber et al. (2021). The results indicated that a minimum of 10 participants would be sufficient to detect statistically significant effects (α = 0.05, power = 0.95). We recruited 15 participants to account for potential dropouts and to increase reliability.

### 2.2 Experimental setup

**FIGURE 1:**
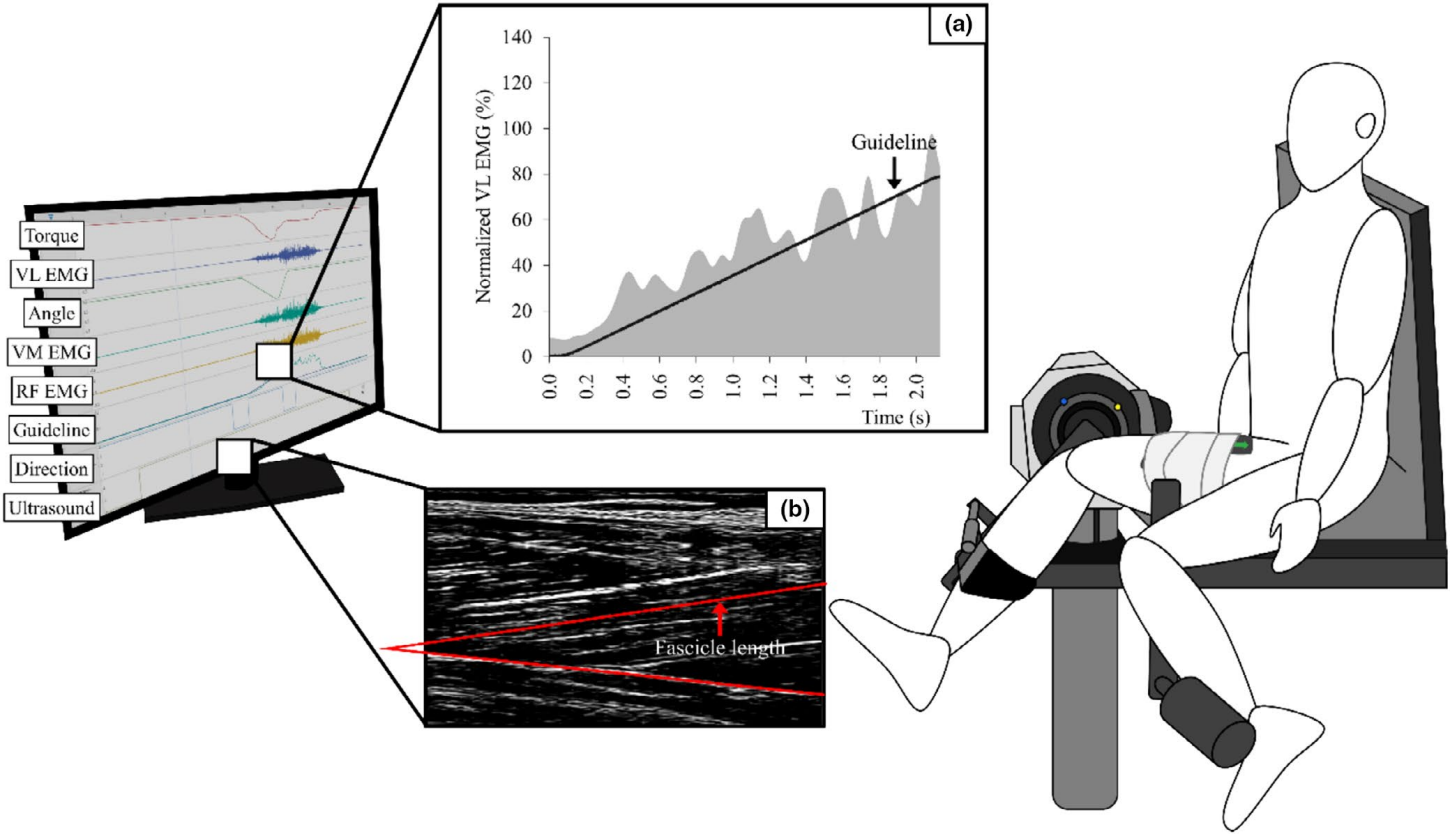
Experimental setting. The participant was seated with the hip flexed at 85°, and the knee joint moved dynamically between 20° and 80° of flexion using an isokinetic dynamometer. The left side of the screen shows a LabChart display panel, which presents all collected physiological signals in real time, including electromyographic (EMG) channel labels corresponding to recorded muscles. (a) An example of a feedback guideline for controlling activation during the stretching phase of ST_80%_‐SC (i.e., target activation of 80% at the end of the stretch). The gray shaded area represents the participant's vastus lateralis (VL) activation, while the black line indicates the target guideline. (b) An ultrasound image of the vastus lateralis captured during stretch‐shortening cycle (SSC), illustrating the muscle fascicle and the superficial aponeuroses. The probe was aligned longitudinally over the VL at the midpoint between the greater trochanter and the lateral epicondyle of the femur to ensure consistent imaging. RF, rectus femoris; VM, vastus medialis.

A dynamometer (CON‐TREX MJ, Multi‐joint module, Swiss TS) assessed knee joint torque. Participants sat with their torso reclined at 85° from the supine position (0°), positioning the hip joint at approximately 95° (relative to full extension, 0°), and the knee joint range of motion was set from 20° to 80° (relative to full knee extension, 0°) (Figure 1).

Muscle activity was measured using a wireless surface electromyography (EMG) system (Trigno Wireless EMG, DELSYS, Boston, MA). EMG electrodes were placed on the muscle bellies of the rectus femoris (RF), vastus lateralis (VL), and vastus medialis (VM), following SENIAM guidelines (www.seniam.org). The skin was prepared by removing hair and exfoliating dead skin cells to reduce resistance and improve signal quality.

The muscle architecture of VL was captured with a real‐time ultrasound imaging system at 80 Hz (LogicScan 128 EXT‐12 kit, TELEMED UAD, Vilnius, Lithuania) with a probe (LV7.5/60/96Z, Samsung Medison, Seoul, Korea). The probe was fixed with compression bandages at the midpoint of a line drawn between the greater trochanter of the femur and the lateral epicondyle of the femur, ensuring stability during voluntary contraction (Nikolaidou et al., 2017).

An analog‐to‐digital converter (PowerLab 16/35, AD Instruments, USA) synchronized all analog signals. The converted signals were stored in a data acquisition program (LabChart Lightening, AD Instruments, Australia) at a sampling rate of 2000 Hz.

### 2.3 Experimental protocol

**FIGURE 2:**
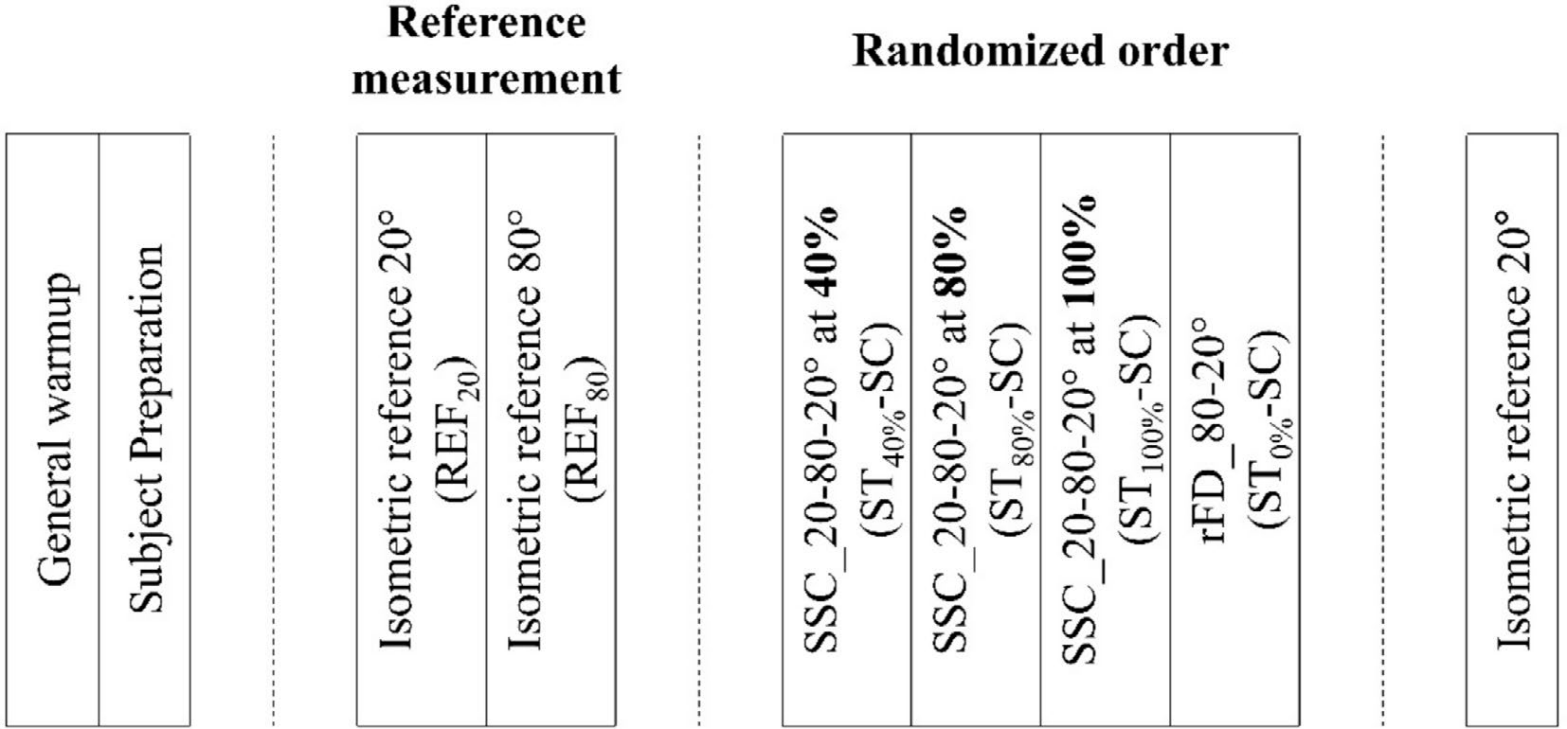
Experimental protocol. Each participant completed a standardized protocol consisting of four phases: (1) a 5‐min warm‐up on a cycle ergometer before testing; (2) subject preparation, including placement of electromyography (EMG) electrodes and the ultrasound probe; (3) reference measurements, including maximal voluntary isometric contraction (MVIC) testing; and (4) stretch‐shortening contraction (SSC) trials under four activation conditions (ST_0%_‐SC, ST_40%_‐SC, ST_80%_‐SC, and ST_100%_‐SC). The order of the four SSC conditions was randomized across participants to minimize order effects. EMG, electromyography; MVIC, maximal voluntary isometric contraction; SSC, stretch‐shortening cycle; ST_0%_‐SC, no activation during the stretch phase; ST_100%_‐SC, maximal activation initiated prior to the stretch; ST_40%_‐SC and ST_80%_‐SC, conditions in which voluntary activation was increased to 40% or 80% of maximal effort by the end of the stretch phase.

Participants completed a 5‐min warm‐up before measurements. Reference fixed‐end knee extension torque was assessed using maximum voluntary isometric contraction (MVC) at 80° (REF_80_) and 20° (REF_20_) knee joint angle (Figure 2). Two trials were performed at each angle, and the average torque was used as the reference. In addition, the mean of the maximum voluntary activation (MVA) of the two REF_80_ trials was used for further EMG normalization (see section Data Acquisition and Analysis).

The SSC trials involved three sequential phases: the knee joint was eccentrically flexed from 20° to 80° at a velocity of 30°/s (stretch phase) with varying activation profiles and immediately extended back to 20° at 120°/s with maximal voluntary effort (shortening phase). Afterward, participants were instructed to maintain maximal effort for at least 2 s at a 20° knee joint angle (fixed‐end phase). There was no pre‐activation before the stretch phase started.

In the two activation‐controlled SSC conditions, voluntary muscle activation started at the beginning of the stretch. It was guided with real‐time feedback to gradually increase from zero to a target activation level of either 40% (ST_40%_‐SC) or 80% (ST_80%_‐SC) MVA that needed to be reached by the end of the stretch phase, followed by maximum voluntary activation from the beginning of the shortening phase. All three phases were performed with maximum effort in the maximal activation SSC condition (ST_100%_‐SC). For the passive stretch condition (ST_0%_‐SC), no muscle activation was allowed during the stretch phase, but the subsequent shortening was performed with maximal effort. The order of the SSC conditions was randomized for each participant to mitigate potential order effects (Figure 2).

### 2.4 Muscle activation control using real‐time EMG feedback

**FIGURE 3:**
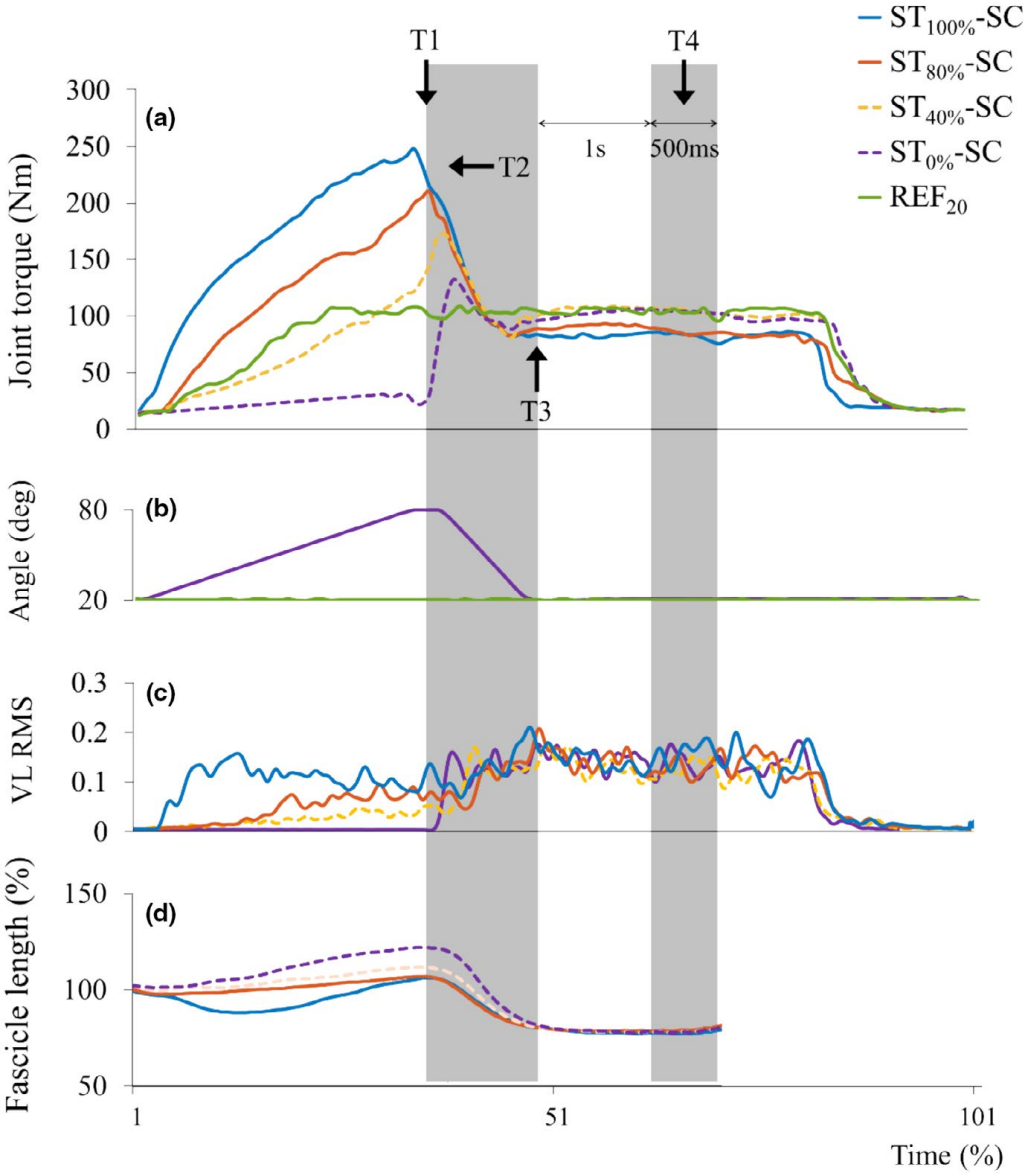
Normalized torque‐time and angle‐time graphs of a representative participant. (a) Joint torque during the stretch‐shortening contraction (SSC). Torque values were normalized to the isometric torque measured at 20° (REF_20_) and are expressed as percentages. The four time points marked on the traces indicate: The end of the stretching contraction (T1), peak torque during the shortening contraction (T2), the end of the shortening contraction (T3), and the steady‐state isometric contraction (T4). (b) Joint angle over time during the SSC task. The first gray shaded area indicates the shortening phase, and the second shaded area represents the measured isometric phase. (c) The Electromyography signals were recorded from the vastus lateralis (VL), vastus medialis (VM), and rectus femoris (RF). VL EMG modulated stretch activation, ensuring controlled activation patterns during the stretch phase. (d) Fascicle length of the VL muscle, normalized to each participant's resting length at 20°.

Real‐time visual feedback was used to help participants gradually increase muscle activation and reach the target activation levels at the end of the stretch phase in activation‐controlled conditions. The feedback was derived from rectified VL EMG signals, processed solely with a 7 Hz low‐pass filter for smoothing (Figure 3). The mean maximum activation from two REF_80_ contractions was the reference (100% MVA) for the activation‐controlled conditions (see section Data acquisition and analysis). Before the experimental trials, participants completed practice trials at 40% MVA to familiarize themselves with the feedback system and activation targets.

During the experimental trials, a trial was initially considered successful if the participant's EMG signal visually aligned with the target guideline. However, trials were repeated if the following occurred: (1) The smoothed EMG signal spiked prematurely or dropped to zero during the stretch phase. (2) The EMG did not reach the target activation range (±20% MVA). (3) Muscle activation ceased at any point during the trial. To prevent fatigue, participants rested for at least 1 min between trials. If participants failed to meet the criteria in two out of 30 attempts, they were excluded from the study. Among the successful trials, the two trials that most closely followed the target guideline were selected and averaged for further analysis.

### 2.5 Data acquisition and analysis

The collected data were processed using LabVIEW 8.5 (National Instruments, Inc., Austin, TX, USA) and MATLAB (MathWorks Inc., USA). EMG signals were band‐pass filtered between 20 and 450 Hz, rectified, and processed to calculate the root mean square (RMS) using a 100 ms window length. The mean maximum of processed EMG signals, recorded during the REF_80_ trials, was used as the reference (100% MVA) for RF, VM, and VL EMG normalization, and EMG data are expressed as a percentage. The average quadriceps activation during the stretch, shortening, and fixed‐end phases, calculated from two successful trials per condition, was measured during the stretching, shortening, and fixed‐end phases.

Discrete torque measurements were taken at the end of the stretching contraction (T1), at peak torque and the respective knee angle during shortening (T2), and at the end of shortening (T3). Fixed‐end torque after the shortening (T4) was calculated as the mean over a 500 ms interval, starting 1 s after the end of shortening. Mechanical work was calculated using the trapezoidal rule by integrating the torque‐time curve during the shortening phase. The SSC effect was assessed by comparing torque and work produced during the shortening phase of the SSC with active stretch (ST_40%_‐SC, ST_80%_‐SC, and ST_100%_‐SC) and those of the passive stretch condition (ST_0%_‐SC) (Equation 1):

(1) $$\mathrm{SSC}\;\text{effect}\;\left(\text{torque},\text{work}\right)\;\left(\%\right)=\frac{\left(\mathrm{SSC}\;\text{trials}-\left({\mathrm{ST}}_{0\%}\mathrm{SC}\right)\right)}{{\mathrm{ST}}_{0\%}\mathrm{SC}}\times 100$$

The history‐dependent effect, either residual force enhancement (rFE) or depression (rFD), was evaluated by comparing the steady‐state isometric torque of all the SSC conditions with that of REF_20_ using the following equation (Equation 2):

(2) $$\text{History}-\text{dependent effect}\;\left(\%\right)=\frac{\left(\text{Torque}-{\mathrm{REF}}_{20}\right)}{{\mathrm{REF}}_{20}}\times 100$$

Ultrasound image data were analyzed using Image‐J software (National Institute of Health, MD, USA) to assess fascicle behavior during dynamic contractions. The fascicle length of VL was measured throughout the stretch and shortening phases to evaluate changes in muscle architecture. Fascicle length was estimated using previously established equations (Finni et al., 2003). Fascicle shortening velocity was calculated from the raw fascicle length, while normalized lengths (to the resting length at 20°) were used for comparison. Fascicle force was estimated as (de Brito Fontana & Herzog, 2016; Ichinose et al., 1997):

(3) $${\text{Fascicle}}_{\text{force}}=\frac{k\times \text{Joint torque}\times {\mathrm{MA}}^{-1}\;}{\cos\left({\text{Fascicle}}_{\text{angle}}\right)}$$

*k* is the physiological cross‐sectional area (PCSA) of the vastus lateralis (Akima et al., 1995), and MA is the moment arm length of VL. Moment arm length was calculated using a third‐order polynomial regression equation fitted to previously reported experimental data (Bakenecker et al., 2019).

Fascicle work during shortening was computed as the area under the force‐length curve, integrating fascicle force and fascicle length over the shortening phase.

(4) $${\text{Fascicle}}_{\text{work}}=\int {\text{Fasiclce}}_{\text{force}}\times {\text{Fasiclce}}_{\text{length}}$$

### 2.6 Statistics

Repeated measures ANOVA was used to compare joint torque, work, muscle activation, and fascicle behaviors (length, force, work, and velocities). Data are presented as mean ± standard deviation (SD). Holm‐Bonferroni corrections were applied for multiple comparisons, with significance at *p* < 0.05. Statistical Parametric Mapping (SPM1D) was employed to analyze the time‐series data, allowing for the independent comparison of torque and normalized fascicle length across conditions over the entire shortening contraction phase (Pataky et al., 2013). Post‐hoc pairwise comparisons were conducted using SPM1D *t*‐tests, and the Holm‐Bonferroni method was applied to control for family‐wise error rates. The analysis was performed with a significance threshold of *α* = 0.05.

## 3 RESULTS

### 3.1 Stretch phase

#### 3.1.1 Muscle activities

The average quadriceps activation during the stretch phase was significantly greater at ST_80%_‐SC (39.7 ± 5.8%) than at ST_40%_‐SC (22.3 ± 5.0%) (*p* < 0.001). Additionally, activation levels were highest in ST_100%_‐SC (61.9 ± 10.5%), showing significantly greater increases compared to both ST_80%_‐SC and ST_40%_‐SC (*p* < 0.001).

#### 3.1.2 Joint torque

At the transition point from the end of the stretching contraction to the shortening contraction (T1), there were no significant differences in torque values between ST_100%_‐SC (240.0 ± 65.5 Nm) and ST_80%_‐SC (219.9 ± 57.2 Nm). Compared to ST_100%_‐SC and ST_80%_‐SC, ST_40%_‐SC (138.8 ± 39.7 Nm) showed significantly lower torque values (*p* < 0.001).

#### 3.1.3 Fascicle behaviors

During MTU stretching, the fascicles were initially slightly shortened and then stretched (Figure 3). The normalized mean fascicle length at the end of the stretch (T1) was shorter for ST_100%_‐SC and ST_80%_‐SC than for ST_0%_‐SC (*p* = 0.005 for both comparisons) (Table 1). However, no significant difference was observed between ST_40%_‐SC and ST_0%_‐SC.

**TABLE 1:** Normalized fascicle length and velocity during shortening contraction.

Conditions	ST_100%_‐SC	ST_80%_‐SC	ST_40%_‐SC	ST_0%_‐SC
Normalized fascicle length (%)				
T1	106.7 ± 11.0^a^	106.8 ± 13.6^a^	112.2 ± 11.6	121.5 ± 10.8
T2	105.4 ± 12.2	105.9 ± 13.7	107.3 ± 12.6	101.6 ± 16.1
T3	80.2 ± 11.4	80.2 ± 9.8	81.1 ± 8.8	82.5 ± 11.8
T4	76.0 ± 11.8	77.3 ± 10.9	76.8 ± 10.5	78.4 ± 9.2
△T1–T3	26.5 ± 9.0	26.6 ± 9.6	31.1 ± 10.7	39.0 ± 17.9
Fascicle shortening velocity (cm/s)				
Maximal velocity	12.6 ± 6.0^a^	12.4 ± 4.8^a^	14.5 ± 4.5^a^	26.3 ± 11.6
Mean velocity	2.5 ± 1.0^a^, ^b^	2.6 ± 1.1^a^	3.1 ± 1.3	3.9 ± 2.0

*Note*: Fascicle length was normalized to each participant's resting length at 20°s. Measurements were taken at four time points (Figure 3): The end of the stretching contraction (T1), maximal torque (T2), the end of the shortening contraction (T3), and during the isometric phase (T4). Fascicle velocity was calculated across the entire shortening phase (△T1–T3). Values are presented as mean ± standard deviation.

Abbreviations: ST_0__%_‐SC, no activation during stretch; ST_40__%_‐SC, voluntary activation increased to 40% of maximal level by the end of the stretching phase; ST_80__%_‐SC, voluntary activation increased to 80% of maximal level by the end of the stretching phase; ST_100__%_‐SC, full activation initiated at the onset of the stretch phase.

^a^ Significant difference with ST_0%_‐SC.

^b^ A significant difference with ST_40%_‐SC.

### 3.2 Shortening phase

#### 3.2.1 Muscle activities

Both ST_100%_‐SC (91.5 ± 22.3%) and ST_80%_‐SC (92.5 ± 20.5%) exhibited significantly greater activation during the shortening phase than ST_40%_‐SC (79.5 ± 17.4%) and ST_0%_‐SC (62.4 ± 19.8%) (*p* < 0.001). Additionally, ST_40%_‐SC showed significantly greater activation than ST_0%_‐SC (*p* < 0.001).

#### 3.2.2 Joint torque and work

**FIGURE 4:**
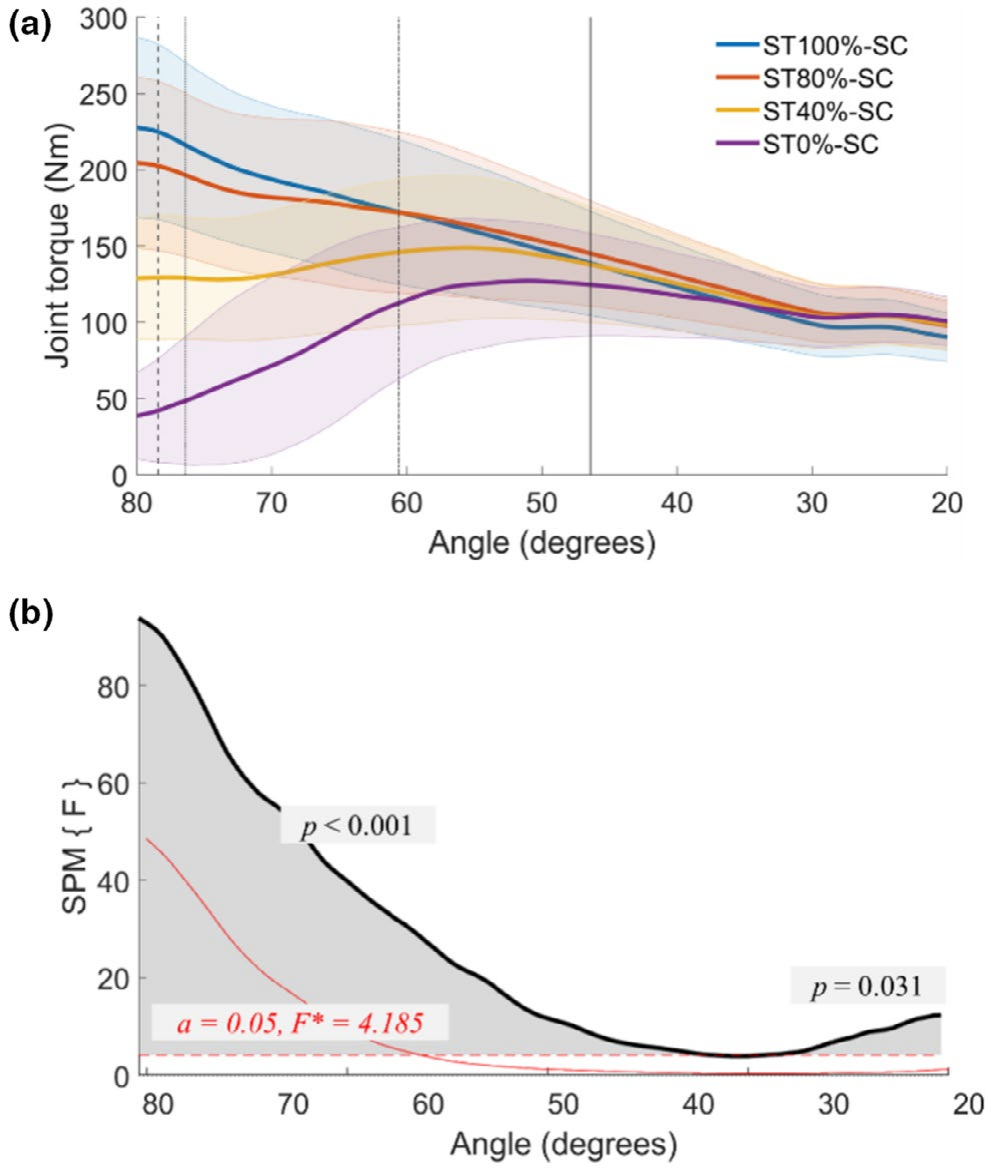
Time‐series analysis of joint torque during the shortening phase using Statistical Parametric Mapping (SPM, bottom). (a) Mean knee joint torque profiles during the shortening phase under each activation condition. The blue line represents ST_100%_‐SC, the orange line represents ST_80%_‐SC, the yellow line represents ST_40%_‐SC, and the purple line represents ST_0%_‐SC. Shaded areas represent the standard deviation around the mean, reflecting variability across participants. Vertical lines represent the joint angles where peak values occurred for each condition: Dashed line (ST_100%_‐SC), dotted line (ST_80%_‐SC), dash‐dotted line (ST_40%_‐SC), and solid line (ST_0%_‐SC). (b) Results of SPM analysis comparing torque trajectories across joint angles. The black curve shows the SPM {F} statistic as a function of joint angle during shortening. The red dashed line represents the critical threshold value (*α* < 0.05; F* = 4.185). The shaded gray area indicates the region where the SPM{F} statistic exceeded the threshold, indicating a statistically significant difference among conditions over that range of joint angles. The SPM{F} statistic is a unitless measure derived from a continuous F‐test applied across the time series (i.e., joint angle), allowing identification of time‐localized differences. For post hoc comparisons of significant differences between conditions, refer to the Results section (SPM time‐series analysis of Torque during shortening). SPM, statistical parametric mapping; SSC, stretch‐shortening cycle; ST_0%_‐SC, no activation during stretch; ST_100%_‐SC, full activation initiated at the onset of the stretch phase; ST_40%_‐SC, voluntary activation increased to 40% of maximal level by the end of the stretching phase; ST_80%_‐SC, voluntary activation increased to 80% of maximal level by the end of the stretching phase.

SPM time‐series analysis of Torque during shortening: From the onset of the shortening phase, torque in ST_40__%_‐SC was lower than in both ST_100__%_‐SC and ST_80__%_‐SC (*p* < 0.001), particularly in the early phase of shortening (80° to 59.5° in ST_100__%_‐SC and 80° to 56.9° in ST_80__%_‐SC) (Figure 4). Compared to ST_0__%_‐SC, torque remained significantly greater throughout the early‐to‐mid shortening phase in all active conditions (*p* < 0.001). Specifically, the torque enhancement persisted up to ~46.9° in ST_40__%_‐SC and ~ 46.2° in ST_100__%_‐SC. In ST_80__%_‐SC, greater torque was observed until 38° (*p* < 0.001).

Toward the end of the shortening phase, torque in ST_100%_‐SC dropped significantly below that of ST_40%_‐SC (*p* = 0.022) and ST_80%_‐SC (*p* = 0.007), particularly in the final phase of shortening (35.0° to 20°). Similarly, torque in ST_100%_‐SC was significantly lower than in ST_0%_‐SC during the late shortening phase (27.7° to 20°, *p* = 0.026).

**FIGURE 5:**
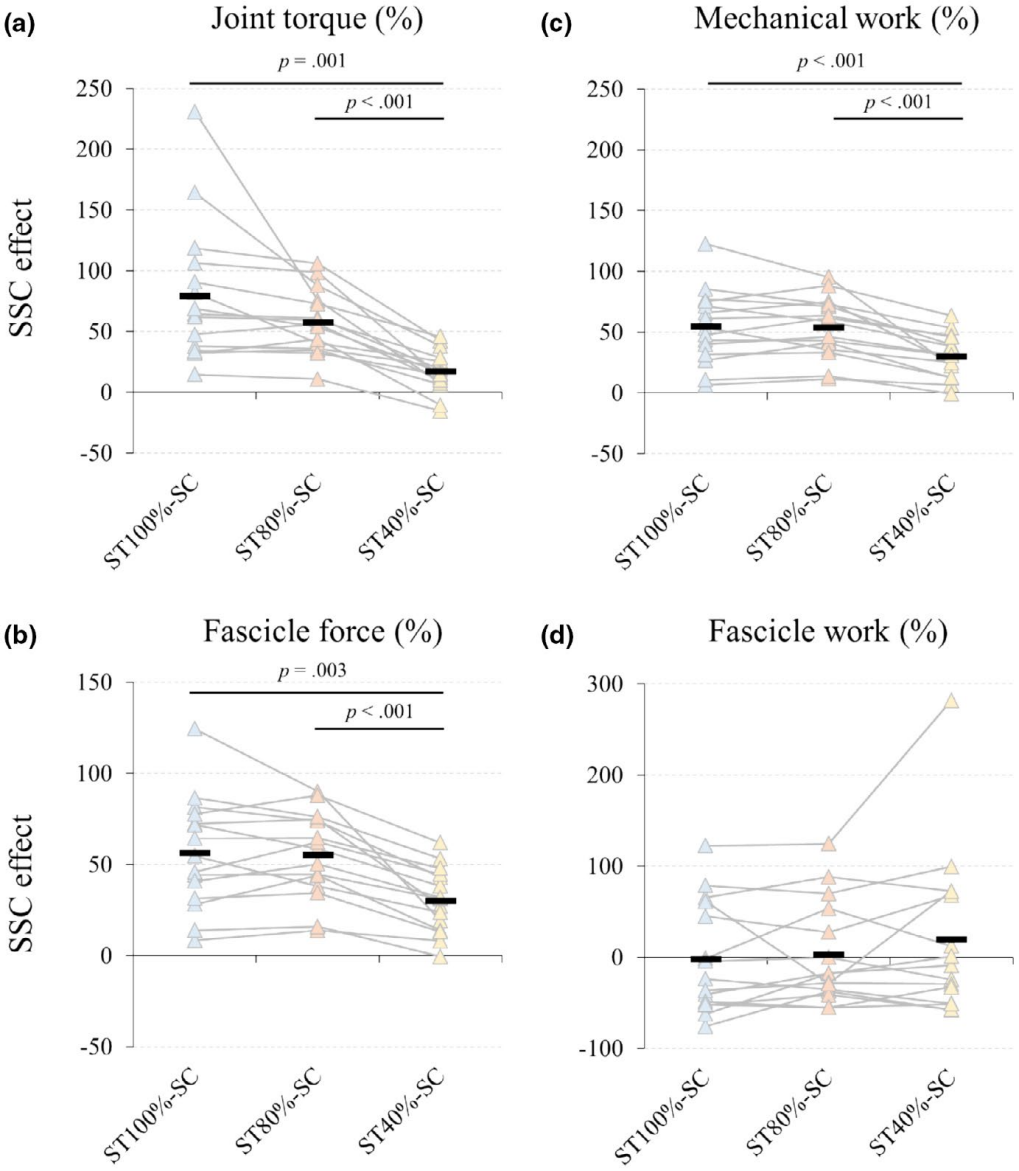
SSC effect of joint torque, fascicle force, and mechanical work during the shortening phase. The SSC effect is expressed as the relative maximal joint torque (a), fascicle force (b), and mechanical work (including fascicle work) (c and d) during shortening contraction compared to the ST_0%_‐SC. (+) indicates an increase relative to ST_0%_‐SC, while (−) indicates a decrease. Each data point represents individual participants, and lines connect repeated measures for the same participant across conditions. Black rectangles indicate the mean values for each condition. The x‐axis labels represent the experimental conditions (ST_40%_‐SC, ST_80%_‐SC, ST_100%_‐SC). ST_0%_‐SC, no activation during stretch; ST_100%_‐SC, full activation initiated at the onset of the stretch phase; ST_40%_‐SC, voluntary activation increased to 40% of maximal level by the end of the stretching phase; ST_80%_‐SC, voluntary activation increased to 80% of maximal level by the end of the stretching phase.

Peak Torque (T2): The peak torque was significantly higher in ST_100%_‐SC (232.9 ± 59.1 Nm) than in ST_80%_‐SC (211.0 ± 56.2 Nm) (*p* = 0.*039*) (Figure 4). Additionally, torque in ST_40%_‐SC (158.8 ± 45.4 Nm) and ST_0%_‐SC (137.6 ± 44.9 Nm) (*p* < 0.001) was shown to be significantly lower than ST_100%_‐SC and ST_80%_‐SC (*p* < 0.001 for all comparisons). ST_40%_‐SC produced higher peak torque compared to ST_0%_‐SC (*p* = 0.*005*). Additional descriptive statistics and 95% confidence intervals are presented in Table S1. The SSC effect, calculated based on torque, was greater at ST_100%_‐SC (*p* = 0.001) and ST_80%_‐SC (*p* < 0.001) than at ST_40%_‐SC (Figure 5a).

The peak torque occurred at 78.4 ± 3.0° and 76.3 ± 6.3° knee joint angles for ST_100%_‐SC and ST_80%_‐SC, respectively, with no significant difference between the two conditions. For ST_40%_‐SC, the peak torque occurred at 60.6 ± 9.7°, and for ST_0%_‐SC it occurred at 46.4 ± 11.1°. The knee joint angles where the peak torque was reached for ST_40%_‐SC and ST_0%_‐SC were significantly delayed compared with those for ST_100%_‐SC and ST_80%_‐SC (*p* < 0.001 for all comparisons). Moreover, ST_40%_‐SC showed a higher peak torque angle than ST_0%_‐SC (*p* = 0.003).

At the end of the shortening phase (T3), all conditions showed significantly lower torque than reference steady‐state isometric torque (107.7 ± 13.0 Nm) (*p* < 0.05). The torque in ST_100%_‐SC (90.2 ± 16.0 Nm) was significantly lower than in ST_80%_‐SC (97.9 ± 16.0 Nm), ST_40%_‐SC (98.6 ± 16.4 Nm), and ST_0%_‐SC (100.6 ± 15.9 Nm) (*p* = 0.005 for all comparison). However, no significant differences were observed among other conditions. Furthermore, the force was a relative reference isometric torque at 20°, which was lower in ST_100%_‐SC (−16.7 ± 7.9%) than in the other conditions (*p* < 0.01 for all comparisons). There were no differences among ST_80%_‐SC (−9.5 ± 6.3%), ST_40%_‐SC (−8.8 ± 7.1%), and ST_0%_‐SC (−6.8 ± 8.3%).

Mechanical work (MTU): Mechanical work in ST_100__%_‐SC (215.5 ± 48.4 J) and ST_80__%_‐SC (216.6 ± 53.3 J) was greater than in ST_40__%_‐SC (185.3 ± 49.7 J) (*p* < 0.001), with no significant differences between ST_100__%_‐SC and ST_80__%_‐SC. Furthermore, the mechanical work in ST_0__%_‐SC (145.2 ± 46.2 J) was significantly lower than in all conditions involving an active stretch (*p* < 0.001 for all comparisons). The SSC effect, calculated from the work, was greater at ST_100__%_‐SC and ST_80__%_‐SC than at ST_40__%_‐SC (*p* < 0.001 for all comparisons) (Figure 5c; see Table S1 for details).

#### 3.2.3 Fascicle behaviors

For normalized fascicle length, no significant differences were observed among conditions (Table 1). All active SSC conditions showed slower maximum fascicle shortening velocity than ST_0%_‐SC (*p* < 0.05) (Table 1). Similarly, the mean fascicle shortening velocity was faster in ST_0%_‐SC than in ST_80%_‐SC and ST_100%_‐SC (*p* < 0.05). Additionally, ST_40%_‐SC exhibited a greater average fascicle shortening velocity than ST_100%_‐SC (*p* = 0.015). No significant differences were observed among the other conditions.

The average fascicle force was significantly greater under SSC conditions compared to the ST_0%_‐SC condition (738.6 ± 236.7 N) (*p* < 0.001). Furthermore, fascicle force was significantly higher at ST_80%_‐SC (1112.4 ± 276.2 N) and ST_100%_‐SC (1109.5 ± 253.5 N) than at ST_40%_‐SC (944.8 ± 256.9 N) (*p* < 0.001). Regarding the SSC effect, both ST_100%_‐SC (56.4 ± 30.7%) and ST_80%_‐SC (55.2 ± 23.7%) showed significantly greater values than ST_40%_‐SC (30.4 ± 17.8%) (*p* < 0.01), whereas no significant difference was observed between ST_80%_‐SC and ST_100%_‐SC (Figure 5; Table S1).

Fascicle work was 26.8 ± 16.9 J for ST_100%_‐SC, 28.7 ± 15.9 J for ST_80%_‐SC, 30.2 ± 17.1 J for ST_40%_‐SC, and 34.3 ± 21.8 J for ST_0%_‐SC condition. No significant differences were observed in fascicle work among conditions. Similarly, regarding the SSC effect compared to passive stretch contraction, no significant differences were found among ST_100%_‐SC (−1.7 ± 61.0%), ST_80%_‐SC (3.1 ± 56.2%), and ST_40%_‐SC (19.6 ± 89.9%).

### 3.3 Steady‐state isometric phase (T4)

#### 3.3.1 Muscle activities

The mean quadriceps activation for each condition was 81.6 ± 18.9% (ST_100%_‐SC), 84.2 ± 18.9% (ST_80%_‐SC), 83.8 ± 18.5% (ST_40%_‐SC), and 79.1 ± 18.4% (ST_0%_‐SC). No significant differences were observed among the conditions.

#### 3.3.2 Joint torque

**FIGURE 6:**
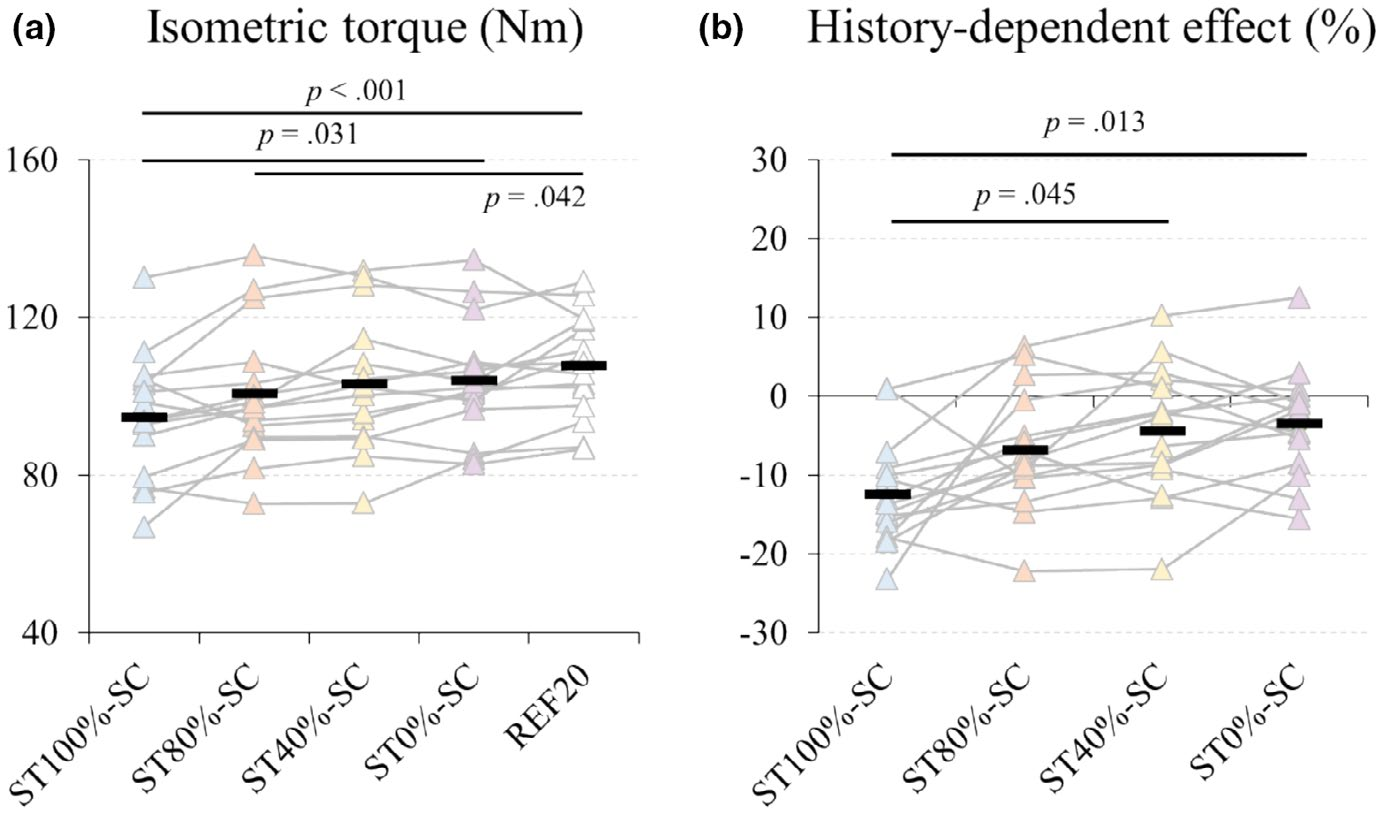
Steady‐state isometric torque and residual force depression following shortening contraction. (a) Steady‐state isometric torque (Nm) measured after the shortening phase. (b) Residual force depression (rFD), calculated as the reduction in steady‐state torque relative to the reference isometric torque at 20° knee flexion (REF_20_). Each data point represents individual participants, and lines connect repeated measures for the same participant across conditions. Black rectangles indicate the mean values for each condition. REF_20_, reference isometric torque at 20°; ST_0%_‐SC, no activation during stretch; ST_100%_‐SC, full activation initiated at the onset of the stretch phase; ST_40%_‐SC, voluntary activation increased to 40% of maximal level by the end of the stretching phase; ST_80%_‐SC, voluntary activation increased to 80% of maximal level by the end of the stretching phase.

The average torque in ST_100%_‐SC was significantly lower than in ST_0%_‐SC (*p* = 0.031) and REF_20_ (*p* < 0.001) (Figure 5; see Table S1 for details). The average torque in ST_80%_‐SC was also significantly lower than in REF_20_ (*p* = 0.042) (Figure 6). No significant differences were found among the other conditions. ST_100%_‐SC showed a 12.3% decrease relative to steady‐state isometric torque, whereas other SSC conditions decreased by ~6.8%. ST_100%_‐SC was significantly different from ST_40%_‐SC (*p* = 0.045) and ST_0%_‐SC (*p* = 0.013).

#### 3.3.3 Fascicle behaviors

No significant differences in normalized mean fascicle length were observed among conditions during the steady‐state isometric phase at T4.

## 4 DISCUSSION

This study examined how progressively increasing voluntary activation during the stretch‐shortening cycle (SSC) stretch phase influences torque generation, mechanical work production, and fascicle dynamics during the subsequent shortening phase. This activation pattern replicates voluntary activation observed in natural human movements. The main findings were (1) all active SSC conditions showed an SSC effect during the subsequent shortening phase, (2) fascicle shortening velocity was lower in all active stretch conditions than in the passive stretch condition during shortening, and (3) high active‐stretch conditions (≥80%) resulted in significantly depressed steady‐state isometric torque after SSCs compared to the reference fixed‐end contraction.

### 4.1 The effect of stretch activation and MTU decoupling in SSC

This study systematically examined voluntary activation levels of knee extensors during the stretch phase of SSC. Torque behavior during the shortening phase depended on the activation level. In ST_80%_‐SC and ST_100%_‐SC, peak torque was reached almost at the onset of shortening and gradually declined thereafter. In contrast, ST_40%_‐SC and ST_0%_‐SC exhibited a rapid rise in activation, producing a brief torque increase before decreasing during the shortening phase.

Consistent with previous findings in in vivo SSC research (Fortuna et al., 2017; Fukutani et al., 2016a; Seiberl et al., 2015), all active SSC conditions exhibited enhanced torque, fascicle force, and mechanical work compared to the reference shortening without active stretch (ST_0%_‐SC). At the end of the stretch (T1), ST_80%_‐SC and ST_100%_‐SC exhibited greater peak torque and shorter fascicle lengths than ST_0%_‐SC, likely reflecting MTU stiffening and slack removal. This placed the fascicles in a more optimal range for force generation (Ichinose et al., 1997) and enabled earlier onset of fascicle shortening. The subsequent decline in torque aligns with force–velocity and force–length relationships (Hill, 1938).

In ST_40%_‐SC, the submaximal activation level at the end of the stretch phase led to a delayed but substantial torque increase during the early‐to‐mid shortening. This likely resulted from the rapid recruitment of additional motor units as muscle activation quickly rose to its maximum at shortening onset. Although fascicle length at the end of the stretch (T1) was ~10% shorter than in ST_0%_‐SC (Table 1), this difference was not statistically significant. However, fascicle shortening velocity was lower in ST_40%_‐SC, suggesting altered muscle‐tendon interactions. These findings indicate that even moderate stretch activation (~40%) can elicit SSC effects.

Despite early torque rises in ST_40%_‐SC and ST_0%_‐SC, their torque output and mechanical work remained lower than in ST_80%_‐SC and ST_100%_‐SC. In particular, ST_0%_‐SC showed nearly double the fascicle shortening velocity of the other conditions, which likely limited torque due to the force‐velocity relationship (Hill, 1938). These differences likely arise from varying degrees of MTU decoupling, which were modulated by the stretch activation profile and influenced subsequent torque production.

Although greater fascicle work would be expected under optimized force‐length‐velocity conditions, no significant differences were observed between conditions. Similarly, Holzer et al. (2024) reported a ~ 35% reduction in gastrocnemius medialis fascicle work during shortening in SSCs compared to pure shortening conditions, suggesting that tendon compliance may have compensated for fascicle shortening (Bohm et al., 2018). This mechanism likely optimizes SSC effects at the joint level while reducing fascicle‐level contributions and thus might reduce metabolic costs. The elastic components of the MTU absorb and release stored energy, reducing the need for active fascicle shortening.

### 4.2 Activation‐dependent SSC effects and residual force depression

Active stretching increases the torque level at the onset of shortening compared to passive‐stretch conditions, but its effect seems transient and concentrated in early shortening (Cronin et al., 2001; Stienen et al., 1978). In this study, however, the SSC effect persisted into the mid‐to‐late shortening phase, particularly at 80% MVC. Two mechanisms may explain this prolonged SSC effect: MTU decoupling, which reduces fascicle shortening velocity and increases force potential (Holzer et al., 2024), and stretch‐induced rFE, which has been shown to persist even at submaximal activation levels (20%–30% MVA) (Dalton et al., 2018; Pinniger & Cresswell, 2007; Seiberl et al., 2012).

At 40% activation, the SSC effect did not persist beyond early shortening, and no residual force depression (rFD) was detected during the steady‐state isometric contraction. In contrast, ST_80%_‐SC maintained the SSC effect throughout the shortening despite the presence of rFD, whereas ST_100%_‐SC exhibited greater rFD during the fixed‐end contraction, with no additional SSC effect beyond the early phase. Our findings indicate that increasing activation beyond 80% MVA did not further enhance SSC effects. EMG data confirmed that both 80% and 100% activation conditions elicited near‐maximal activation during shortening, yet no additional benefit was observed at 100% conditions (ST_100%_‐SC). One possible explanation is that inhibitory mechanisms may have limited force production to protect the MTU from excessive strain (Aagaard et al., 2000; Westing et al., 1991). However, our EMG data did not provide direct evidence of inhibition, such as firing rate reductions. Alternatively, higher activation conditions (e.g., ST_100%_‐SC) may have increased mechanical stress during the stretch phase, leading to greater rFD and potential reductions in motor unit recruitment during shortening.

Although rFD is generally linked to mechanical work performed during shortening (De Ruiter et al., 1998; Edman, 1996), this assumption has been recently challenged (Raiteri et al., 2024). For example, submaximal voluntary fixed‐end tibialis anterior dorsiflexion findings suggest that rFD can occur independently of fascicle/MTU work. Instead, motor unit recruitment during shortening may better predict rFD (Raiteri et al., 2024). Accordingly, a lower number of active motor units at the beginning of shortening may explain the absence of rFD in passive‐stretch and low active‐stretch conditions, whereas significant rFD was observed following SSCs with high active‐stretch conditions (Figure 6). These findings highlight the interaction between muscle activation, mechanical work, and residual force depression, emphasizing that increasing activation beyond 80% MVA does not provide additional SSC benefits but may contribute to greater rFD.

### 4.3 Limitation

The shortening velocity in this study was significantly lower than the typical speeds observed in jump performance (~572°/s) (Jo & Lee, 2023; Papaiakovou, 2013). This discrepancy may have influenced the observed SSC effects since slower shortening velocities are linked to increased rFD (Herzog et al., 2000).

Additionally, higher activation levels were associated with increased rFD, indicating that activation‐dependent force modulation is a key factor in SSC dynamics. While rFD is well‐documented under controlled conditions, its impact on movement execution and neuromuscular efficiency in dynamic tasks remains unclear. Future research should explore this relationship to better understand its functional significance in movement.

Furthermore, fascicle force and work estimations were based on net joint torque scaled to the vastus lateralis using literature‐derived moment arm and PCSA values. We used a regression equation provided by previous studies, in which moment arms were measured at knee joint angles ranging from 40° to 90° (Bakenecker et al., 2019). Although this approach is grounded in prior work, it does not account for inter‐individual anatomical differences (Bakenecker et al., 2019; Krevolin et al., 2004), and therefore, the absolute force and work values should be interpreted with caution. Nevertheless, given that the same assumptions were applied consistently across all conditions, we believe the relative comparisons between conditions remain meaningful.

## 5 CONCLUSION

This study confirms that gradually increasing voluntary activation during the stretch without isometric pre‐activation, resembling activation patterns in natural human movements, induces the SSC effect in large in vivo human muscles. The observed SSC effect may be attributed to the muscle activation level during stretch‐inducing pre‐activation and pre‐load for the following shortening phase that triggers the decoupling behavior between the MTU and fascicles. While greater stretch activation enhanced the early‐phase SSC effect, excessive stretch activation led to more pronounced torque loss toward the end of shortening, with significant rFD after the SSC. These findings highlight the importance of the integrated mechanisms of SSC for a better understanding of natural human movements.

## AUTHOR CONTRIBUTIONS

Iseul Jo, Wolfgang Seiberl, and Hae‐dong Lee conceived and designed the research. Iseul Jo performed the experiments, analyzed the data, and prepared the figures. Iseul Jo, Wolfgang Seiberl, and Hae‐dong Lee interpreted the results of the experiments. Iseul Jo and Hae‐dong Lee drafted the manuscript. Iseul Jo, Wolfgang Seiberl, and Hae‐dong Lee edited and revised the manuscript and approved the final version.

## FUNDING INFORMATION

This work was supported by the National Research Foundation of Korea Grant funded by the Korean Government (NRF‐2023S1A5B5A19093381).

## CONFLICT OF INTEREST STATEMENT

The authors declare no conflicts of interest.

## ETHICS STATEMENT

The Yonsei University Institutional Review Board approved the study protocol (7001988‐202405‐HR‐2275‐02). All participants provided written informed consent prior to participation.

## Supporting information


Table S1.

## Data Availability

All relevant data supporting the findings of this study are included in the article and are available upon reasonable request from the corresponding author.
