# Supplementary material for: Modulation of stretch activation influences the stretch‐shortening cycle effect in in vivo human knee extensors
Source: Physiol Rep. 2025 May 15;13(10):e70377. doi: 10.14814/phy2.70377 (PMC12081825; doi:10.14814/phy2.70377)
Supplement: Supplementary file 1 — Table S1. [file PHY2-13-e70377-s001.docx]

**Table S1**. Descriptive statistics and 95% confidence intervals (CI) for variables showing significant differences between conditions. Mean, standard deviation (SD), and 95% CI (lower and upper bounds) are reported. The 95% CIs were calculated based on the sample mean and standard error.

Abbreviations: ST_0%_-SC, no activation during stretch; ST_40%_-SC, voluntary activation increased to 40% of maximal level by the end of the stretching phase; ST_80%_-SC, voluntary activation increased to 80% of maximal level by the end of the stretching phase; ST_100%_-SC, full activation initiated at the onset of the stretch phase.

|  |  | **Mean** | **SD** | **Lower 95% CI** | **Upper 95% CI** |
| --- | --- | --- | --- | --- | --- |
| *Shortening contraction* | | | | | |
| **Peak torque (Nm)** | ST_100%_-SC | 232.9 | 59.1 | 200.2 | 265.7 |
|  | ST_80%_-SC | 211.0 | 56.2 | 179.8 | 242.1 |
|  | ST_40%_-SC | 158.8 | 45.4 | 133.7 | 184.0 |
|  | ST_0%_-SC | 137.6 | 44.9 | 112.7 | 162.5 |
| **Mechanical work (J)** | ST_100%_-SC | 215.5 | 48.4 | 188.7 | 242.3 |
|  | ST_80%_-SC | 216.6 | 53.3 | 187.1 | 246.1 |
|  | ST_40%_-SC | 185.3 | 49.7 | 157.7 | 212.8 |
|  | ST_0%_-SC | 145.2 | 46.2 | 119.6 | 170.8 |
| **Fascicle force (N)** | ST_100%_-SC | 1109.5 | 253.5 | 969.1 | 1249.9 |
|  | ST_80%_-SC | 1112.4 | 276.2 | 959.5 | 1265.4 |
|  | ST_40%_-SC | 944.8 | 256.9 | 802.5 | 1087.0 |
|  | ST_0%_-SC | 738.6 | 236.7 | 607.5 | 869.6 |
| *SSC effect* | | | | | |
| **Peak torque (%)** | ST_100%_-SC | 79.1 | 57.8 | 47.1 | 111.1 |
|  | ST_80%_-SC | 57.9 | 26.5 | 43.2 | 72.6 |
|  | ST_40%_-SC | 17.3 | 17.4 | 7.7 | 27.0 |
| **Mechanical work (%)** | ST_100%_-SC | 54.6 | 30.4 | 37.7 | 71.4 |
|  | ST_80%_-SC | 54.0 | 24.9 | 40.2 | 67.8 |
|  | ST_40%_-SC | 30.1 | 18.1 | 20.1 | 40.2 |
| **Fascicle force (%)** | ST_100%_-SC | 56.4 | 30.7 | 39.4 | 73.4 |
|  | ST_80%_-SC | 55.2 | 23.7 | 42.1 | 68.4 |
|  | ST_40%_-SC | 30.4 | 17.8 | 20.5 | 40.2 |
| *Fascicle shortening velocity* | | | | | |
| **Maximal velocity (cm/s)** | ST_100%_-SC | 12.6 | 6.0 | 9.3 | 15.9 |
|  | ST_80%_-SC | 12.4 | 4.8 | 9.7 | 15.1 |
|  | ST_40%_-SC | 14.5 | 4.5 | 12.1 | 17.0 |
|  | ST_0%_-SC | 26.3 | 11.6 | 19.9 | 32.8 |
| **Mean velocity (cm/s)** | ST_100%_-SC | 2.5 | 1.0 | 1.9 | 3.1 |
|  | ST_80%_-SC | 2.6 | 1.0 | 2.0 | 3.2 |
|  | ST_40%_-SC | 3.1 | 1.3 | 2.4 | 3.8 |
|  | ST_0%_-SC | 3.9 | 2.0 | 2.8 | 5.0 |
| *Steady-state isometric contraction* | | | | | |
| **Isometric torque (Nm)** | ST_100%_-SC | 94.8 | 16.0 | 86.0 | 103.6 |
|  | ST_80%_-SC | 100.8 | 17.2 | 91.2 | 110.3 |
|  | ST_40%_-SC | 103.3 | 17.1 | 93.8 | 112.8 |
|  | ST_0%_-SC | 104.1 | 14.9 | 95.8 | 112.3 |
|  | REF_20_ | 107.7 | 13.0 | 100.5 | 115.0 |
| **History-dependent effect (%)** | ST_100%_-SC | -12.3 | 6.8 | -16.1 | -8.6 |
|  | ST_80%_-SC | -6.8 | 7.7 | -11.0 | -2.5 |
|  | ST_40%_-SC | -4.3 | 8.3 | -8.9 | 0.3 |
|  | ST_0%_-SC | -3.4 | 6.9 | -7.2 | 0.4 |
